# First comparative proteomic and *in vitro* behavioral study of *Echinococcus granulosus* metacestodes in *Felis catus*

**DOI:** 10.3389/fvets.2025.1546420

**Published:** 2025-09-02

**Authors:** Andrea Maglioco, Vanesa V. Miana, María Pía Valacco, Facundo A. Agüero, María Laura Gertiser, Héctor G. Avila, Melisa S. Barbery Venturi, Oscar Jensen, Alejandra Y. Juárez Valdez, Elio A. Prieto González, Alicia G. Fuchs

**Affiliations:** ^1^Centro de Altos Estudios en Ciencias Humanas y de la Salud (CAECIHS), Universidad Abierta Interamericana (UAI), Buenos Aires, Argentina; ^2^Consejo Nacional de Investigaciones Científicas y Técnicas (CONICET), Buenos Aires, Argentina; ^3^CEQUIBIEM, Facultad de Ciencias Exactas y Naturales, UBA, Buenos Aires, Argentina; ^4^Centro de Zoonosis Sarmiento, Provincia de Chubut, Argentina; ^5^Laboratorio Provincial de Zoonosis de San Juan, San Juan, Argentina; ^6^Instituto Nacional de Parasitología “Dr Mario Fatala Chaben”, ANLIS-Malbrán, Buenos Aires, Argentina

**Keywords:** cystic echinococcosis, larva, proteomics, metabolism, vitality

## Abstract

*Echinococcus granulosus* sl is the etiological agent of cystic echinococcosis affecting livestock and humans worldwide. *Felis catus* contributes to the dispersion of parasite eggs and the connection between wild and domestic populations. The potential larval development in the intermediate host or its capacity to develop the worm stage in the definitive host is not disclosed. Protein expression profiles may reveal parasite adaptations to the intermediate host. This study presents, for the first time, a comparative analysis of the *in vitro* behavior, cytogenetics, and Liquid Chromatography-mass/mass spectrometry (LC-MS/MS)-based molecular profile of two *Echinococcus granulosus* s.s. (G1 genotype) metacestodes isolated from two naturally infected, unrelated *Felis catus* hosts without FIV. Protein abundance index (emPAI) analysis showed distinct proteomic signatures. Metacestode from Cat # 1 was predominantly characterized by proteins involved in glucose intermediary metabolism, energy production, Adenosin- tri- phosphate (ATP)-dependent contractile filaments, antigenic proteins, and DNA repair, suggesting a molecular profile potentially more adapted to survival or development within the definitive host. In contrast, metacestode from Cat # 2 predominantly expressed proteins associated with inflammation and membrane components rich in heparan sulfate, suggesting reduced viability or invasiveness. Despite similarities in *in vitro* parameters, including cytogenetics, primary parasite cell growth, and protoscolex development, mass spectrometry analysis revealed differences in protein expression patterns between the two metacestodes. In conclusion, this study highlights molecular markers that may contribute to understanding the adaptive strategies and pathogenic potential of *E. granulosus* s.s. metacestodes. Host diversity and parasite metabolic profile may provide new insights into parasite behavior, virulence, and host–parasite interactions.

## 1 Introduction

*Echinococcus granulosus* sensu lato (*E. granulosus*) is the etiological agent of cystic echinococcosis (CE) and, based on cox-1 mitochondrial DNA sequences, is classified into several genotypes: *E. granulosus* s.s. (G1–G3) is found mainly in sheep and buffalo, *E. equinus* (G4) in horses, *E. ortleppi* (G5) in cattle, *E. canadensis* (G6/7) in pigs, camels, and goats, and *E. canadensis* (G8/10) in cervids. *E. felidis* has only been identified in lions (1). Further analysis performed in the nad5 gene polymorphism discriminated diversity in G1 and G3 genotypes (2). Haplotypes based on cox1 and nad2 (3), as well as cox2 and nad6 in G1/G3 and G5 genotypes, showed greater genetic variability in G1 compared to G5 (4). Restriction fragment length polymorphism (RFLP) analysis of the nad1 gene was able to distinguish G1–G3 from G6–G7 in the hyperendemic region (5). The distribution of CE genotypes in humans reflects the regional presence of the infected intermediate host. Human infection has been identified with genotypes G1/G3 and G6/G7 (6), while infections with G5, G8, and G10 (7, 8) are not frequent. A meta-analysis of *E. granulosus* genotype revealed that *E. granulosus* s.l. has a global distribution and is particularly prevalent in South America; the most reported genotypes globally were G1/G3 (47.3%), G7 (15.3%), G5 (14.6%), and G6 (13.3%) (9). In Argentina, the G1 genotype has also been detected in pigs and cattle, and it represents the most common genotype associated with human infection (10, 11). A study conducted on human patients from Turkana reported genotype-specific patterns related to sex, age, and cyst pathogenesis (5).

The infection of the intermediate hosts is determined by the parasite's life cycle, which involves two different hosts. The hermaphrodite worm inhabits the intestine of the definitive host, a member of the *Canidae* family, and produces infective embryos, known as oncospheres. The worm can survive for ~4 years in the definitive host, releasing oncospheres into the environment through the feces. These oncospheres persist in the soil until they are ingested by an intermediate host, typically an ungulate animal, or by an accidental host (12), and a survey on oncosphere genotype provides spatial-temporal epidemiological data (13). Risk factors for human infection include living with infected dogs, poor hygiene conditions, and proximity to uncontrolled slaughterhouses (14). For ungulate livestock, key risk factors include intensive breeding practices and close contact with infected dogs (15). *Felis catus*, as an accidental intermediate host, may become infected through grooming behavior or by rolling on contaminated ground. The oncosphere can adhere to the cat's fur and legs, turning the animal into a passive mechanical vector capable of carrying oncospheres to humans and livestock within the peridomestic area. Furthermore, *Felis catus* may serve as a link between the wild and domestic transition cycle, as suggested by Abdullah et al., (16). Moreover, the domestic-wild transition interface may influence the genetic and epigenetic adaptability of the parasite, potentially increasing or decreasing its infectivity and thereby affecting the persistence of the parasite's life cycle.

In the digestive system of the intermediate host, oncospheres become activated and penetrate the intestinal barrier into the systemic circulation. The embryos migrate to the liver, where they develop into the larval stage, or metacestode, within 2 to 3 months. The metacestode has two membranes: an outer, acellular laminar membrane that interfaces with the host tissues, and an inner cellular proliferative membrane. The proliferative membrane immersed in hydatid fluid (HF) generates embryos asexually, protoscolex (pe) (17). These pe evolve within the cyst, transitioning from an invaginated to an evaginated form and developing up to two proglottids. They are characterized by strobilus with hooks and suckers and are surrounded by an external acellular tegument. Secondary metacestode originates when the laminal membrane is broken, releasing pe into the host's body. However, the primary developmental pathway for pe is to become an adult worm, when *Canidae* eats infected raw viscera. One of the distinguishing features of the *E. granulosus* metacestode, in contrast to other *Cestodes*, is its ability to develop pe into a juvenile scolex (18), exhibiting a variety of cell types and physiological characteristics across different developmental stages (19, 20).

The parasite–host relation in *E. granulosus* infection is complex. The metabolic diversity of the metacestode may trigger differential inflammatory responses. One of the hallmark features of the metacestode is the induction of a chronic fibrotic inflammatory reaction that surrounds the cyst's laminar membrane. The metacestode establishes a close interaction with the host, modulating immune response and releasing molecules necessary for lipid transport to the parasite (21, 22). Among the major antigenic molecules secreted into the HF, the polymeric lipoprotein antigen B (EgAgB), characterized by its high polymorphism, comprises five subfamilies: AgB1 to AgB5. It is probably secreted in its monomeric molecular form, 8 kDa (23). Other antigens are Ag5 (24) or P29 (25), EgSeverin, and Eg14-3-3 (26) or hexokinase (27).

*In vitro* cell culture models are widely used to study tumor cells, aggressiveness, where the cell growth rate is often correlated with invasion and metastasis. In contrast, stem cells and embryonic cell cultures are primarily employed to study cell differentiation processes. For parasitological studies, metacestode or parasite isolated cells can be cultured *in vitro* to explore parasite evolution (28). These cultures are viable tools for pre-clinical pharmacology testing (29, 30), the identification of novel antigenic proteins (27, 31), and the investigation of various physiological characteristics (32). Moreover, cell culture is necessary to perform cytogenetics analysis (33), which can reveal chromosome integrity and completion of cell metaphase. The adaptation of *E. granulosus* to different hosts and environmental conditions may require specific mechanisms such as alternative splicing (34). However, those adaptive mechanisms may also result in chromosome instability, including deletions or insertions, presence of double minutes or extra chromosomal material (35). Encystation of the metacestode and the survival of pe depend on several physicochemical characteristics and these are regulated at the transcriptional level through the upregulation of antioxidant molecules, metabolic enzymes, and kinases (36). The ability to transition between developmental stages may be driven by genomic plasticity, potentially leading to chromosome variability.

Previous studies performed by our group, on the EGPE cell line, derived from *E. granulosus* ss/G1 pe, developed cystic colonies in agarose without pe sprouting. Cytogenetic analysis revealed hyperploid cells showing termini-terminal junction (33). In additional studies using the EGPE cell line, we observed differential antigen expression depending on the duration of cell culture (37). Furthermore, patients' sera showed reactivity against four *E. granulosus'* histones (31). The survival of pe in the intermediate host and its development into a worm in the definitive host depend on the host's immune response (22, 38) and the parasite's virulence. Cyclical fluctuation in parasite virulence factors can overcome the host's immunological resistance, thus favoring the establishment of infection (39).

Cases of CE in *Felis catus* have been reported accidentally in several regions, including Uruguay (40), Italy (41), Iraq (42), Australia (16), and Chile (43), Russia (44), Turkey (45), and our report of cases in Patagonia, Argentina (46, 47). These findings have epidemiological relevance, as domestic cats may act as passive vectors or accidental intermediate hosts, highlighting the need for strategic control measures to prevent transmission to humans and livestock. In this study, we present results from two *E. granulosus* s.s /G1 metacestodes found in naturally infected *Felis catus* from different rural towns in Patagonia, Argentina. Both animals were treated in a veterinary clinic and showed abdominal cysts (46, 47).

This study explores the biological characteristics of metacestodes recovered from *Felis catus* to provide insights into the behavior and potential to complete the parasite life cycle. Analyses included *in vitro* behavior, cytogenetics, and protein expression patterns in vesicles randomly sampled from metacestodes from two unrelated cats. These parameters were assessed as indicators of metabolic diversity and developmental potential.

## 2 Materials and methods

### 2.1 Parasite handling

Parasite vesicles were collected either during surgery (Cat #1) or at necropsy (Cat #2) into sterile 50 mL Falcon tubes containing 15.5 mM HEPES and 10 mM EDTA (pH 7.4). Samples were transported on ice in a secure cold container to the laboratory.

### 2.2 Primary cell culture

The sample from Cat #1 was processed immediately upon arrival. HF was centrifuged at 1,200 rpm for 8 min, and the resulting sediment, containing pe and cells, was washed 5 times with PBS buffer. The sediment was diluted and divided into two parts.

#### 2.2.1 Primary cell culture without trypsin activation

Half of the sample were seeded into a six- wells cultured plate containing medium 199 (Sigma) supplemented with 10% of filtrated HF (through 0.45 and 0.25 μm syringe filter), 1 mM sodium pyruvate (sodium salt extra pure, Anhedra, Beijing, China), and 78 μg/mL β-mercaptoethanol (Merck, Darmstadt, Germany) and penicillin/ streptomycin. The medium was adjusted to pH 7.9. Cells were incubated in a humidified incubator at 37°C with 5% CO_2_/ air for 7 days. After this period, the culture medium was centrifuged at 4,000 rpm for 10 min, and the sediment was collected. Cell viability was assessed using the trypan blue exclusion method, showing 90% of viability. A total of 1 × 10^5^ cells/ ml were seeded in 96-well plates in medium 199 supplemented with 10% fetal bovine serum (FBS, Internegocios, Argentina) instead of HF for cell growth assay (33).

#### 2.2.2 Cell culture with trypsin activation

The remaining half of the sediment was incubated with 2.5% trypsin in PBS at 37°C for 80 min. After incubation, trypsin was diluted, and the sample was centrifuged at 4,000 rpm. The resulting sediment was seeded into a six-well culture plate with the same medium described above and incubated for 1 week. After incubation, cell viability was assessed (95%), and 1 × 10^5^ cells/ ml were seeded into 96-well plates as described above.

#### 2.2.3 Cell growth assay

Cell viability and growth were assessed using the trypan blue exclusion method every 2 days, for 14 days, in triplicate.

### 2.3 Protoscolex primary culture

The entire pe isolated from the Cat # 2 sample was washed five times with PBS and then activated with 2.5% trypsin in PBS at 37°C for 80 min. Afterward, trypsin was diluted and the pe were seeded into 90 mm Petri dishes and incubated under the same culture conditions described above until the pe disassembled. Fresh culture medium was added weekly. Culture was maintained for 1 month and observed every 3 days using an inverted stereoscopic microscope. Images were captured when changes in pe development were observed.

### 2.4 Cytogenetics

#### 2.4.1 Cell culture

Cells from Cat # 1, without trypsin incubation, were cultured in a six-well plate as described above, using medium supplemented with 10% HF for 7 days. After the first passage, the cells were further cultured for an additional week, in culture medium supplemented with 10% FBS.

#### 2.4.2 Chromosome harvest

When the cells reached 80% confluence, 0.1 μg / mL colcemid was added to each well. Cells were incubated at 37°C in a 5% CO_2_ incubator for 2 h. The supernatant was discarded, and the cells were treated with 0.25% trypsin (Gibco, New York, NY, USA) for ~2 min. Trypsin activity was neutralized by adding medium supplemented with 10% FBS. The cell suspension was transferred to 15 mL tubes (Corning) and centrifuged at 200 × *g* for 10 min. Cells were subjected to hypotonic treatment using 10 mL of 0.075 M KCl at 37°C for three cycles. During each cycle, cells were gently shaken by oscillation for 2 min and incubated at 37°C for 15 min. Final hypotonic shock was applied for 20 min at 37°C. Then, cells were centrifuged at 200 x *g* for 5 min at 25°C, the supernatant was discarded, and the pellet was resuspended in 10 mL of cold, freshly prepared Carnoy's fixative (methanol/ acetic acid; 3:1).

#### 2.4.3 Slide preparation

A fixative was slowly added while gently resuspending the pellet to avoid cell clumping. The suspension was mixed using a Pasteur pipette, centrifuged at 200 × g for 5 min, and resuspended twice in Carnoy's fixative. The cell suspension was dropped from a height of 40 cm onto clean slides angled at 45°. The slides were air-dried at room temperature and stored in dust-free boxes until analysis.

#### 2.4.4 Chromosome staining: Giemsa and C-banding

The technique modified from Sumner (48) and Kostmann et al. (49) was applied. Slides were dried at 60°C for 1 h, then incubated in 0.2 N HCl for 30 min, followed by 5% Ba(OH)_2_ at 45°C for 5 min. A final incubation was carried out in 2 × saline sodium citrate at 60°C for 70 min. Metaphase chromosomes were stained with 0.4% Giemsa solution in tap water for 15 min, followed by rinsing with distilled water.

#### 2.4.5 Metaphase observation and scoring

Slides were then air-dried before being observed at 400 and 1,000 x magnification in a Zeiss AxioPlan microscope. Chromosomes stained with G and C bands were analyzed for their sizes and centromere position, and heterochromatic regions were identified as C-band. Images were captured, processed, and analyzed with ADOBE Photoshop CS2 software.

### 2.5 Mass spectrometry

#### 2.5.1 Samples

Several vesicles from both cats were stored dried upon receipt, individually preserved in liquid nitrogen for subsequent LC-MS/MS analysis.

#### 2.5.2 Protein extraction and sample preparation

Proteins were obtained from one parasite vesicle from each *Felis catus* (Cat # 1 and Cat # 2), stored in liquid nitrogen, and thawed for processing. They were broken mechanically in lysis buffer (8 mmol/ L CHAPS, MP Biomedicals, 10 mmol/L Tris–HCl, pH 8, 2 mmol / L EDTA, 0.1% B-mercaptoethanol, MP Biomedicals, and 1/100 protease inhibitor cocktail, Sigma-Aldrich). Samples were frozen and thawed five times in lysis buffer until the vesicle was almost disintegrated, leaving only scattered filaments resting on the back. The samples were spun down at 10,000 × *g*, and the proteins obtained in the supernatant were measured using the Bradford assay. A total of 30 μg proteins from each sample were loaded into preparative electrophoresis gels (acrylamide–bis-acrylamide) and allowed to migrate 1 cm into the separating gel (Supplementary Figure 1). Protein bands were visualized by colloidal Coomassie blue-staining.

#### 2.5.3 Sample processing

Mass Spectrometry analysis was performed at CEQUIBIEM, the Core Facility of the Faculty of Exact Sciences, University of Buenos Aires/IQUIBICEN CONICET, National Research Council, Argentina. Protein bands containing the whole protein extract from Coomassie blue-stained SDS-PAGE gels were excised and were sequentially washed and detained with 50 mM ammonium bicarbonate, 25 mM ammonium bicarbonate, 50% acetonitrile, and 100% acetonitrile; reduced and alkylated with 10 mM DTT and 20 mM yodoacetamide and in-gel digested with 100 ng Trypsin (Promega v5111) in 25 mM ammonium bicarbonate overnight at 37°C. Peptides were recovered by elution with 50% acetonitrile and 0.5% trifluoroacetic acid, including brief sonication, and further concentrated by speed–vacuum drying. Samples were resuspended in 15 μL of water containing 0.1% formic acid, desalted using C18zip tips (Merck-millipore), and eluted in 10 μl of H_2_O: ACN: FA (40:60:01%).

#### 2.5.4 nanoHPLC and mass spectrometry data acquisition

The digests were analyzed using nanoLC-MS/MS in a Thermo Scientific Q Exactive Mass Spectrometer coupled to a nanoHPLC EASY-nLC 1,000 (Thermo Scientific). For LC-MS/MS analysis, ~2 μg of peptides were loaded onto the column and eluted for 120 min using a reverse-phase column (C18, 2 μm, 100 A, 50 μm × 150 mm), Easy-Spray Column PepMap RSLC (P/N ES801), suitable for separating protein complexes with a high degree of resolution. The flow rate used for the nanocolumn was 300 nL 1,000-1, and the solvent range was from 7% B (5 min) to 35% (120 min). The gradient duration was 120 min. Solvent A was 0.1% formic acid in water, whereas B was 0.1% formic acid in acetonitrile. The injection volume was 2 μL. The MS equipment has a high collision dissociation cell (HCD) for fragmentation and an Orbitrap analyzer (Thermo Scientific, Q Exactive). A voltage of 3.5 kV was used for Electro Spray Ionization (Thermo Scientific, EASY-SPRAY). XCalibur 3.0.63 (Thermo Scientific) software was used for data acquisition. Full-scan mass spectra were acquired in an Orbitrap analyzer. The scanned mass range was 400–1,800 m/z, at a resolution of 70,000 at 400 m/z, and the 12 most intense ions in each cycle were sequentially isolated, fragmented by higher-energy collision dissociation, and measured in an Orbitrap analyzer. Peptides with a charge of +1 or with an unassigned charge state were excluded from fragmentation for MS2.

#### 2.5.5 Analysis and protein identification

Q Exactive raw data was processed using the Proteome Discoverer software (version 2.1.1.21, Thermo Scientific) and searched against the *E. granulosus, Felis* catus, and virus from the feline immunodeficiency virus (FIV) sequences database UniProt (50) with trypsin specificity and a maximum of one missed cleavage per peptide. Carbamidomethylation of cysteine residues was set as a fixed modification, and oxidation of methionine was set as a variable modification. Proteome Discoverer searches were performed with a precursor mass tolerance of 10 ppm and product ion tolerance of 0.05 Da. Protein hits were filtered for at least one high-confidence peptide matched with a maximum protein and peptide false discovery rate of 1%, calculated by using a reverse database strategy. The mass spectrometry instrument was calibrated with commercial calibration standards and standardized controls for protein digestion, and nanoHPLC runs and instrument performance were performed before and after every sample.

### 2.6 Protein classification

Protein hits identified by at least two unique peptide sequences and two or more peptide spectrum matches (PSMs) were classified as either “shared” if the proteins were present in both samples or “non-shared” if they were present in only one sample. Proteins were then grouped and classified.

#### 2.6.1 Shared proteins

Clusters for shared proteins were membrane proteins; proteins involved in glycosylation; calcium-binding; contractile filaments; cytoskeletal proteins and microfilaments; nuclear proteins; proteins involved in glucose metabolism; mitochondrial proteins; proteins involved in lipid metabolism; protein synthesis; proteins involved in proteolysis; signal transduction and nervous system; oxide-reduction and detoxification; antigenic proteins, and, uncharacterized proteins.

#### 2.6.2 Non-shared proteins

Clusters for non-shared proteins were glucose metabolism, mitochondrial proteins, antigens, nuclear proteins, protein glycosylation, microfilaments, contractile filaments, protein synthesis, oxide-reduction, proteolysis, signal transduction, nervous system, lipid metabolism, membrane proteins, and uncharacterized proteins.

### 2.7 Analysis of percent identity

For protein entries annotated in the database with similar names but different UniProt accession identifiers, sequence alignment was performed using BLASTp to determine percent identity.

### 2.8 Statistical analysis of data

#### 2.8.1 Cell growth assay

Viable metacestode-derived cells were cultured, and proliferation was evaluated. Each data point represents the mean of a triplicate sample; mean values ± SD were calculated. Student's *t*-test was applied at each time point to compare the cell number between cultures with and without trypsin activation.

#### 2.8.2 Proportional analysis of shared proteins

The emPAI% parameter (exponentially modified protein abundance index), calculated using Proteome Discovered software, was employed to estimate and compare protein expression levels in both samples.

##### 2.8.2.1 Estimation of protein relative abundance

To estimate the relative abundance of identified proteins in both metacestodes, Cat # 1 and Cat # 2, the emPAI index was calculated by Sequest using protein identification data. PAI was calculated using the number of observable peptides and the number of observed parent ions. To calculate the number of observable peptides per protein, proteins were digested *in silico*, and the obtained peptide masses were compared with the scan range of the mass spectrometer. PAI was converted to exponentially modified PAI (emPAI), equal to 10 PAI minus one, which is proportional to protein content in a protein mixture. The equation emPAI/Σ (emPAI) was used to calculate the protein content in mol % (emPAI%) (51, 52).

##### 2.8.2.2 Proportional calculation of shared proteins

To determine the 100% of protein content for each sample, Cat # 1 and Cat # 2, the sum of all emPAI values was used. Ferritin was excluded from this total due to its high value, which could skew the proportion of other proteins.

##### 2.8.2.3. Heatmap visualization

Proportional protein values were plotted using a heatmap. To visualize the low proportion, a log_10_ value was applied.

##### 2.8.2.4 Statistical analysis of proportional protein values

Graphical visualization methods using protein classification in functional clusters might not highlight differences in proteins with the extreme values, either very high or very low proportions. Then, a statistical comparison was performed using the chi-squared test. The X^2^ test was conducted using the “Social science statistics” (53), with significance levels of *p* < 0.001 and *p* < 0.05.

#### 2.8.3 Proportional analysis of non-shared protein

Non-shared proteins were evaluated according to the number of proteins found in each sample (100%) from Cat # 1 or 2. Then “X^2^” test was used to analyze statistical differences. The level of significance was *p* < 0.05.

## 3 Results

### 3.1 Metacestode morphological studies and *in vitro* behavior

**Figure 1:**
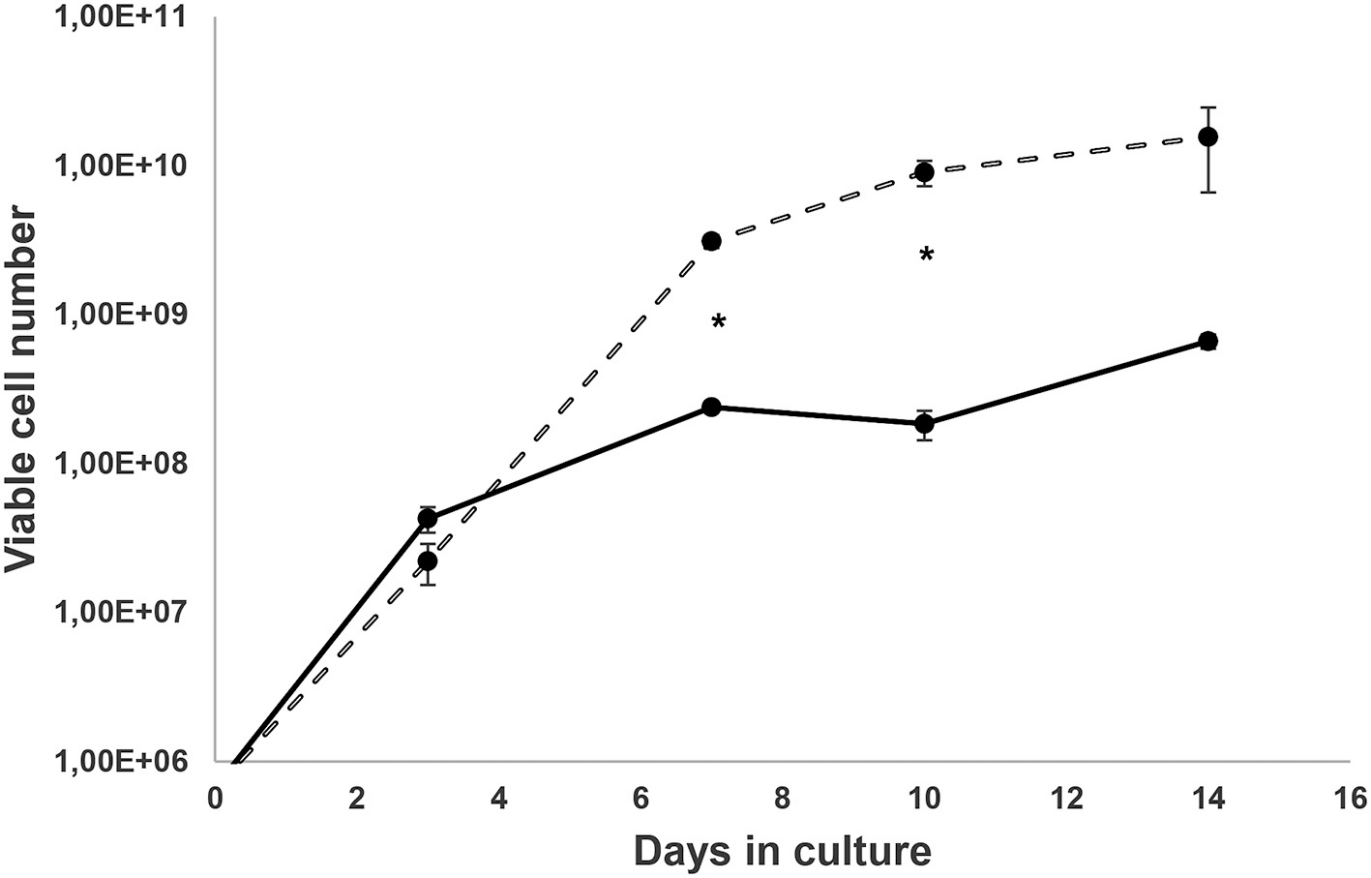
*Ex vivo* metacestode's isolated cell growth. Cells from the larval stage of Cat # 1 *Echinococcus granulosus* ss/G1 were cultured for a week in medium 199 and 10% of hydatid fluid, and then cells were diluted in medium 199 with 10% fetal bovine serum as described elsewhere (33). Cells from vesicles were obtained without trypsin 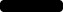 and with trypsin activation 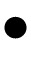

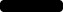
. Points are the media ± SD of viable cell numbers from three wells, and (*) shows significant differences (*p* < 0.05) evaluated by Student's *t*-test.

**Figure 2:**
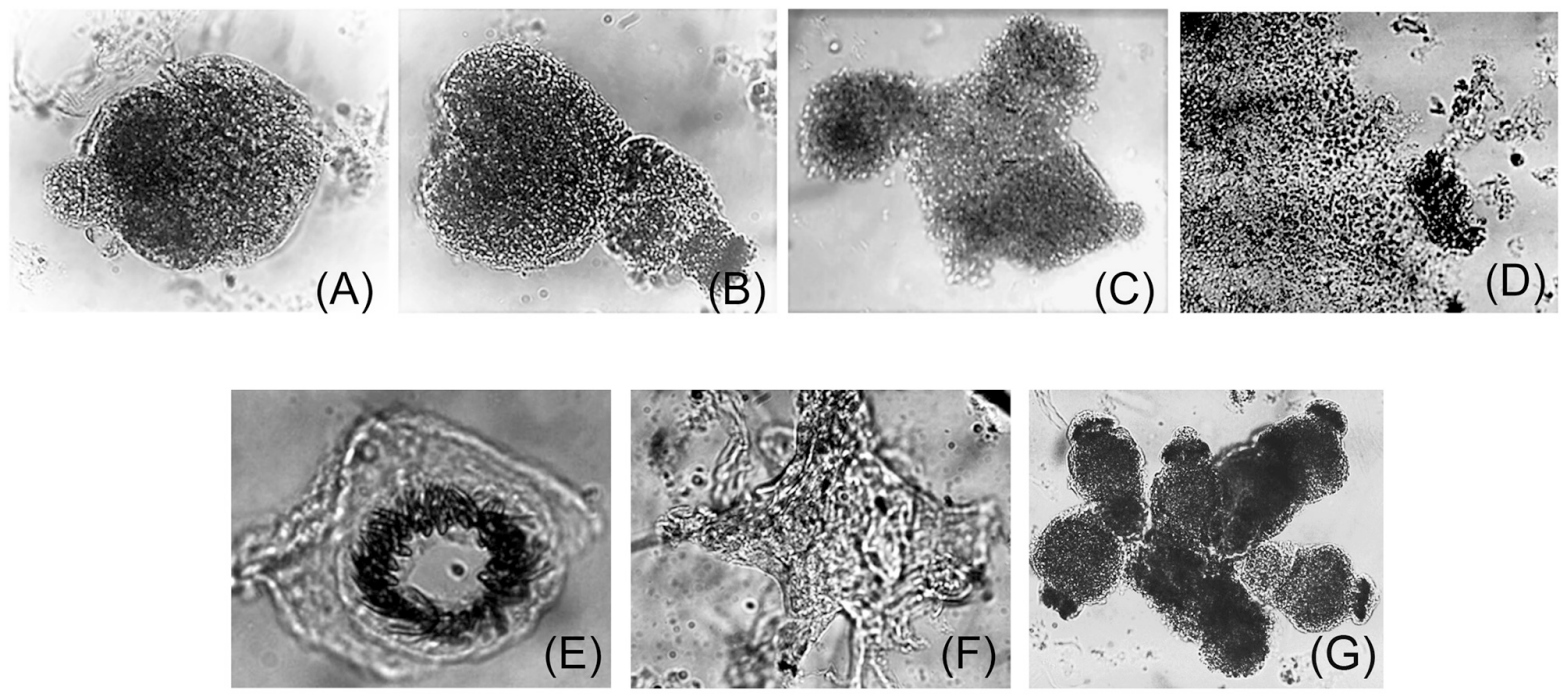
*Ex vivo* protoscolex development in culture. Trypsin-treated protoscolex (pe) from Cat # 2 *Echinococcus granulosus* s.s./G1, larval stage were seeded in tissue culture plates and incubated in a CO_2_ incubator for 1 month with medium 199, 10% SFB as described (33). **(A–C)** show pe from time 0 (T0) to the 15^th^ day. Afterward, the scolex structure was lost **(D)** and the hook was released to the media **(E)**. **(F)** shows a membrane surrounded by cells. **(G)** Shows the grouped protoscolex before its disassembly.

Samples from *Felis catus* were free from FIV infection, as confirmed by LC-MS/MS studies. Cells derived from Cat # 1 were cultured, and cell proliferation is presented in Figure 1. *Ex vivo* and *in vitro* development of pe, obtained from Cat # 2, is shown in Figure 2. Trypsin treatment did not disassemble the structure of the pe and metacestode membranes (Figure 2A); however, it did stimulate primary cell culture growth (Figure 1). In contrast, cells from the parasite's vesicle that were not trypsin-treated exhibited significantly slower growth. The described pe evolution in culture was observed up to stage 4 (Figures 2A–D, F). At this stage, the pe lost their hook and their distinctive structure and the strobilus differentiation (Figure 2E). Isolated cells adhered to the unfolded membrane (Figure 2F). Interestingly, during pe incubation *in vitro*, pe(s) temporally clustered together before disassembly (Figure 2G).

### 3.2 Cytogenetic analysis

**Figure 3:**
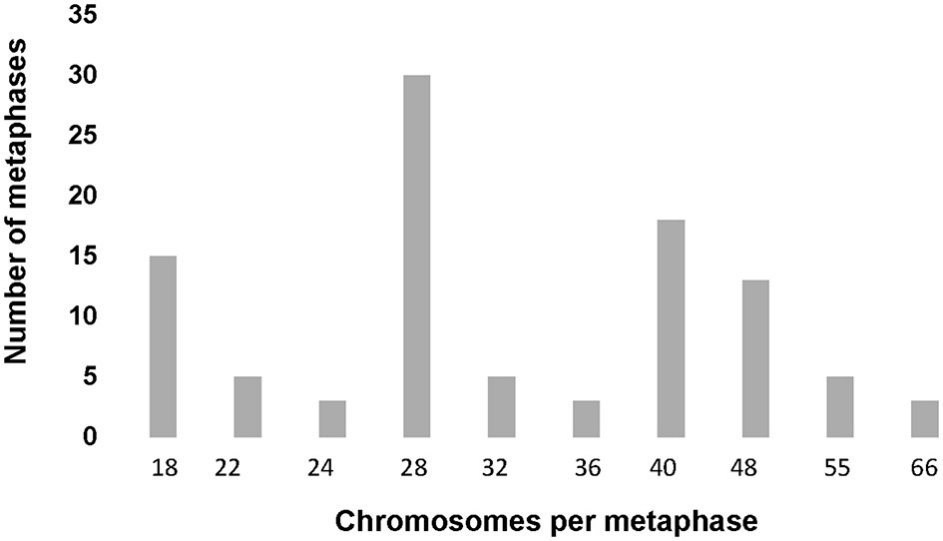
Modal chromosome number histogram. One hundred metaphases from the second passage primary cell culture from *Echinococcus granulosus* s.s./G1 (Cat # 1) were Giemsa and C-band stained. Chromosomes were analyzed and classified under 1,000x magnification in a transmitted light microscope. The modal class of chromosomes was 28 and 40, and 15% of the metaphases showed a diploid number of chromosomes, according to Rausch et al. (71).

**Figure 4:**
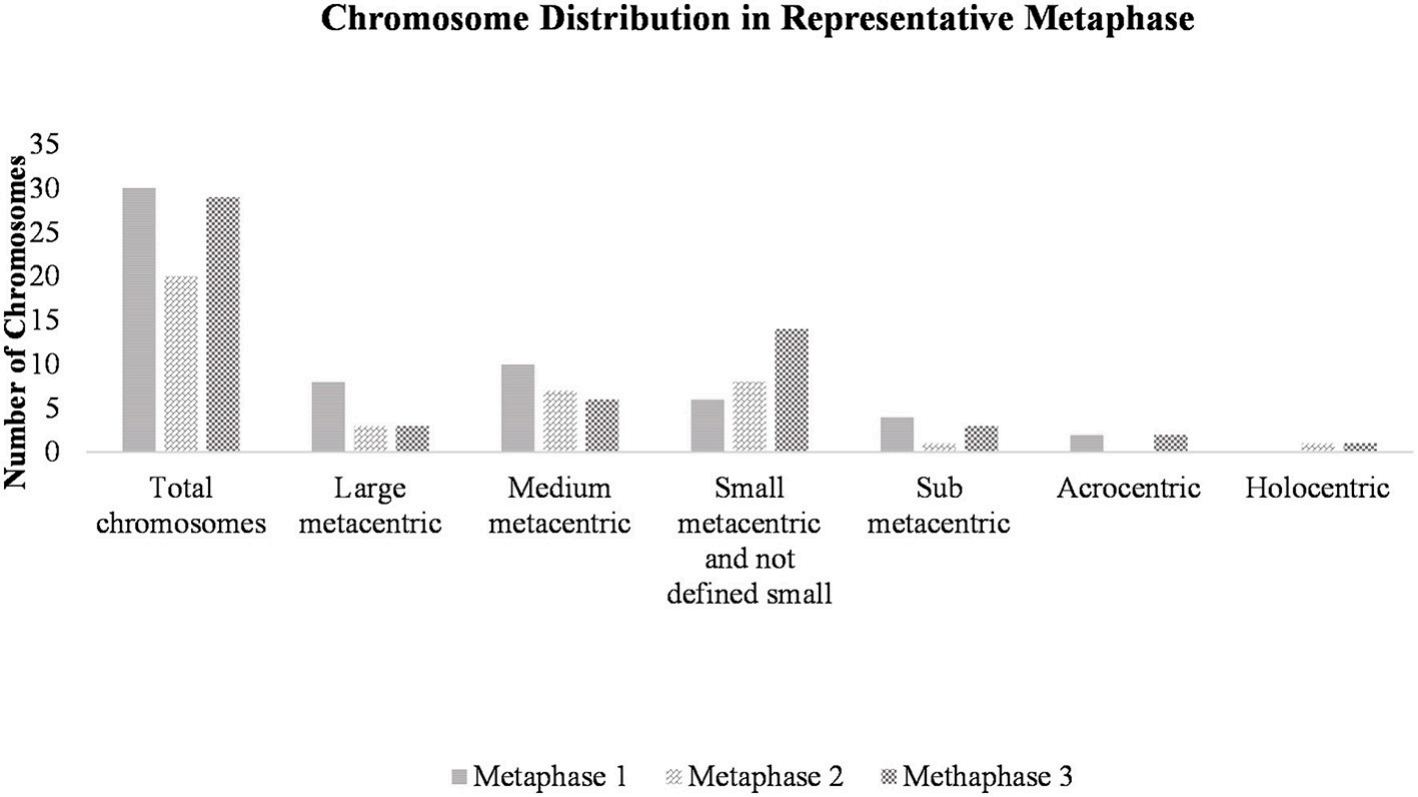
Chromosome distribution in a representative metaphase. Cell suspensions were subjected to hypotonic treatment and dropped onto clean slides at a 45° angle, followed by air drying. After staining with Giemsa, three representative metaphases were selected and observed at 1,000x magnification using a stereoscopic microscope. Chromosomes were counted and classified.

Heteroploid metaphases were detected even in primary cultures derived from the hydatid cysts. Chromosomes prepared with G- and C-bandings were documented photographically and studied for their sizes and the position of the centromere (Supplementary Figure 2). A total of 100 Giemsa-stained metaphases were examined to determine the chromosome count per cell and to evaluate the modal chromosome distribution number (Figure 3). The size and centromere position distribution, based on G- and C – band profiles, of three representative metaphases are shown in Figure 4.

A noteworthy observation was the frequent termini-terminal association of chromosomes, occurring in ~7% of metaphases. Long arrays of chromosomes associated through their extreme regions, telomere sequences, were found. Three of those associations visualized using C- banding are illustrated in Supplementary Figure 2F.

### 3.3 Mass spectrometry analysis

A total of 222 host-contaminating proteins were identified in Cat # 1 and 287 in Cat # 2. Proteins identified as originating from *E. granulosus* numbered 340 in Cat # 1 and 246 in Cat # 2. Among the metacestode proteins, 225 were shared between the two samples. In Cat # 1, 115 proteins were unique (non-shared), while in Cat # 2, 21 unique proteins were detected (Supplementary Tables 1, 2).

#### 3.3.1 Shared proteins

**Figure 5:**
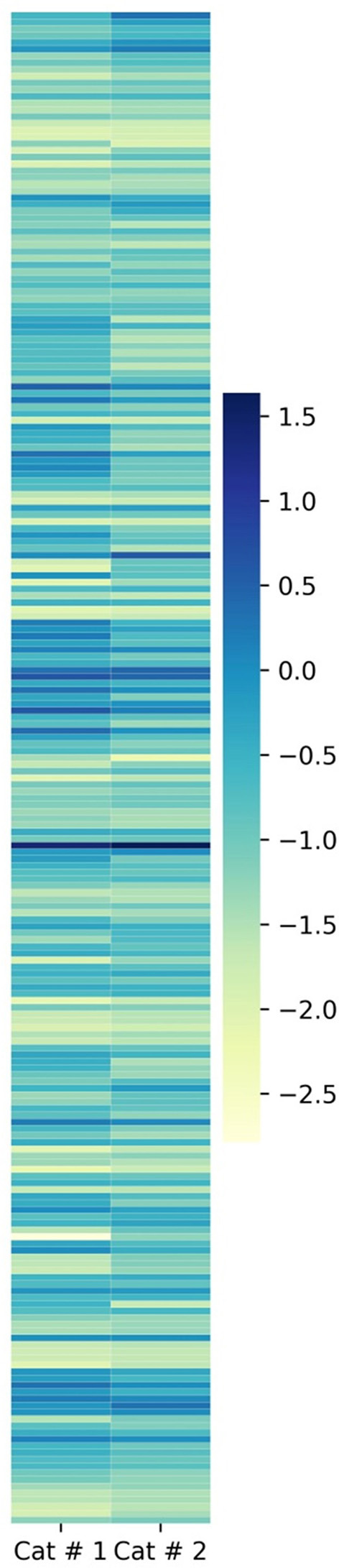
Heatmap of metacestodes shared proteins. Proportional representation of total metacestode shared proteins. Proportion was obtained by empAI (emPAI % = emPAI*100 / Σ (emPAI(s)- ferritin emPAI). To visualize the low proportional value, a log_10_ value was applied.

The total emPAI for shared proteins was 21.4303 in Cat # 1 and 37.3253 in Cat # 2. Since ferritin accounted for a high proportion in both cats (Supplementary Table 3), relative protein abundance analysis was performed by subtracting the emPAI values of ferritin. After this correction, the total emPAI was 8.6977 for Cat # 1 and 6.2757 for Cat # 2. Differences in relative protein abundance were assessed using a heatmap (Figure 5) and evaluated for statistical significance at two levels, *p* < 0.05 and *p* < 0.001, using the chi-squared test.

##### 3.3.1.1 Subcellular localization

###### 3.3.1.1.1 Membrane proteins and proteins involved in glycosylation

**Figure 6:**
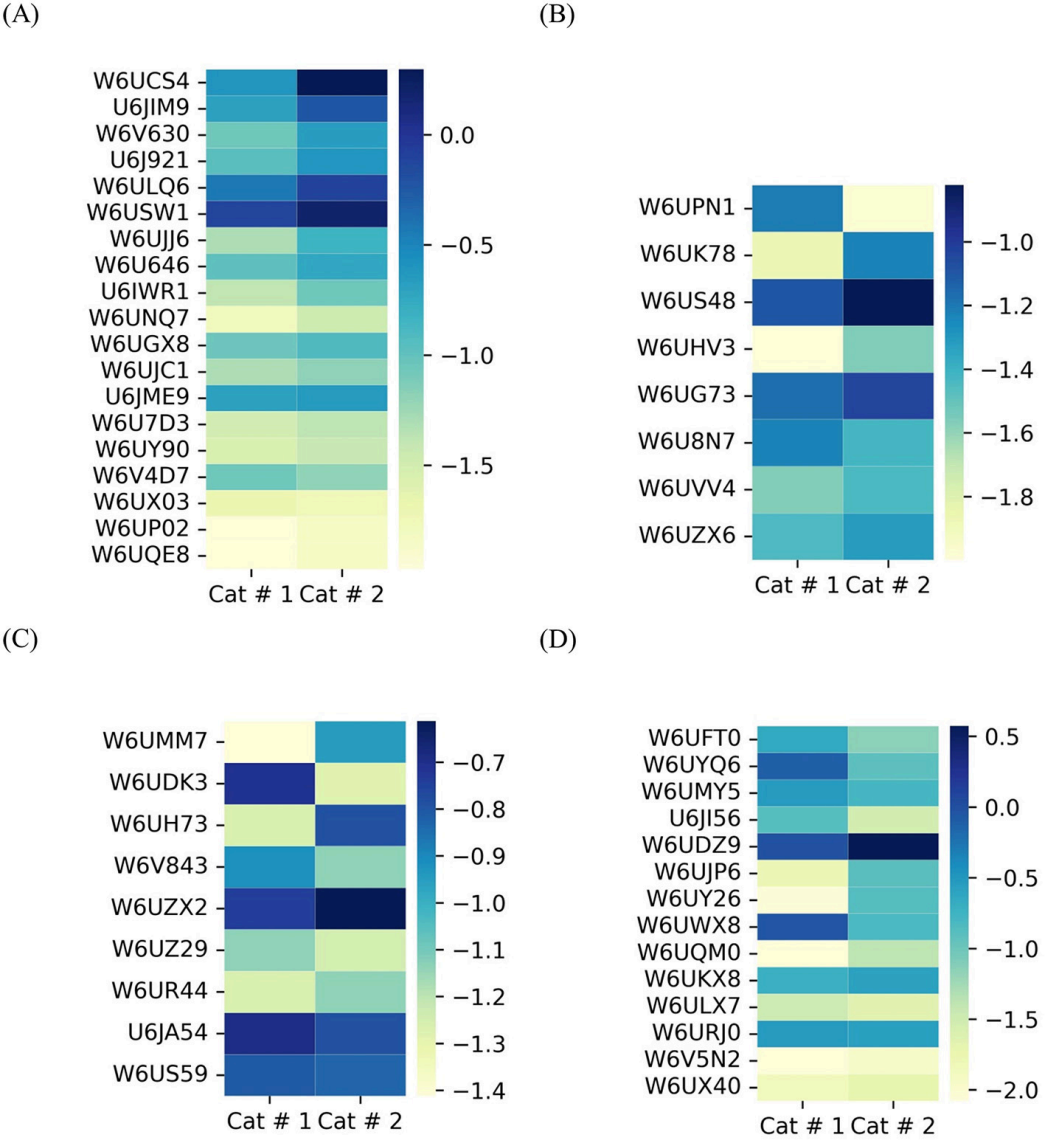
Heatmap of shared metacestode proteins classified by clusters. The proteins are organized from top to bottom according to X^2^ test results. To visualize low proportions, the log_10_ value was applied. **(A)** Membrane proteins, *p* < 0.001: from W6UCS4 to W6U646; *p* < 0.05: U6IWR1 and W6UNQ7; and without significant differences: W6UGX8 to W6UQE8. **(B)** Proteins involved in glycosylation. *p* < 0.001: from W6UPN1 to W6US48; *p* < 0.05: W6UHV3; and without significant differences: from W6UG73 to W6UZX6. **(C)** Proteins involved in calcium binding. *p* < 0.001: from W6UMM7 to W6UH73; *p* < 0.05: W6V843 and W6UZX2; and without significant differences: W6UZ29 to W6US59. **(D)** Contractile filaments. *p* < 0.001: from W6UFT0 to W6UQM0; *p* < 0.05: W6UKX8 and without significant differences: from W6ULX7 to W6UX40.

Membrane proteins (Figure 6A) and proteins involved in glycosylation (Figure 6B) showed higher relative abundance in Cat # 2, with 10 out of 19 and 3 out of 8 proteins, respectively. Although the proportion of calcium-binding proteins was evenly distributed between samples, W6UZX2, a calcium-binding protein, had a significantly higher relative proportion in Cat # 2 (Figure 6C).

###### 3.3.1.1.2 Contractile filaments

They were classified based on their energy requirements. Cat # 1 showed higher proportional values for proteins associated with high energy demand, whereas Cat # 2 showed higher proportions for those associated with lower energy requirement (Figure 6D, Supplementary Table 3).

###### 3.3.1.1.3 Cytoskeletal proteins and filaments

**Figure 7:**
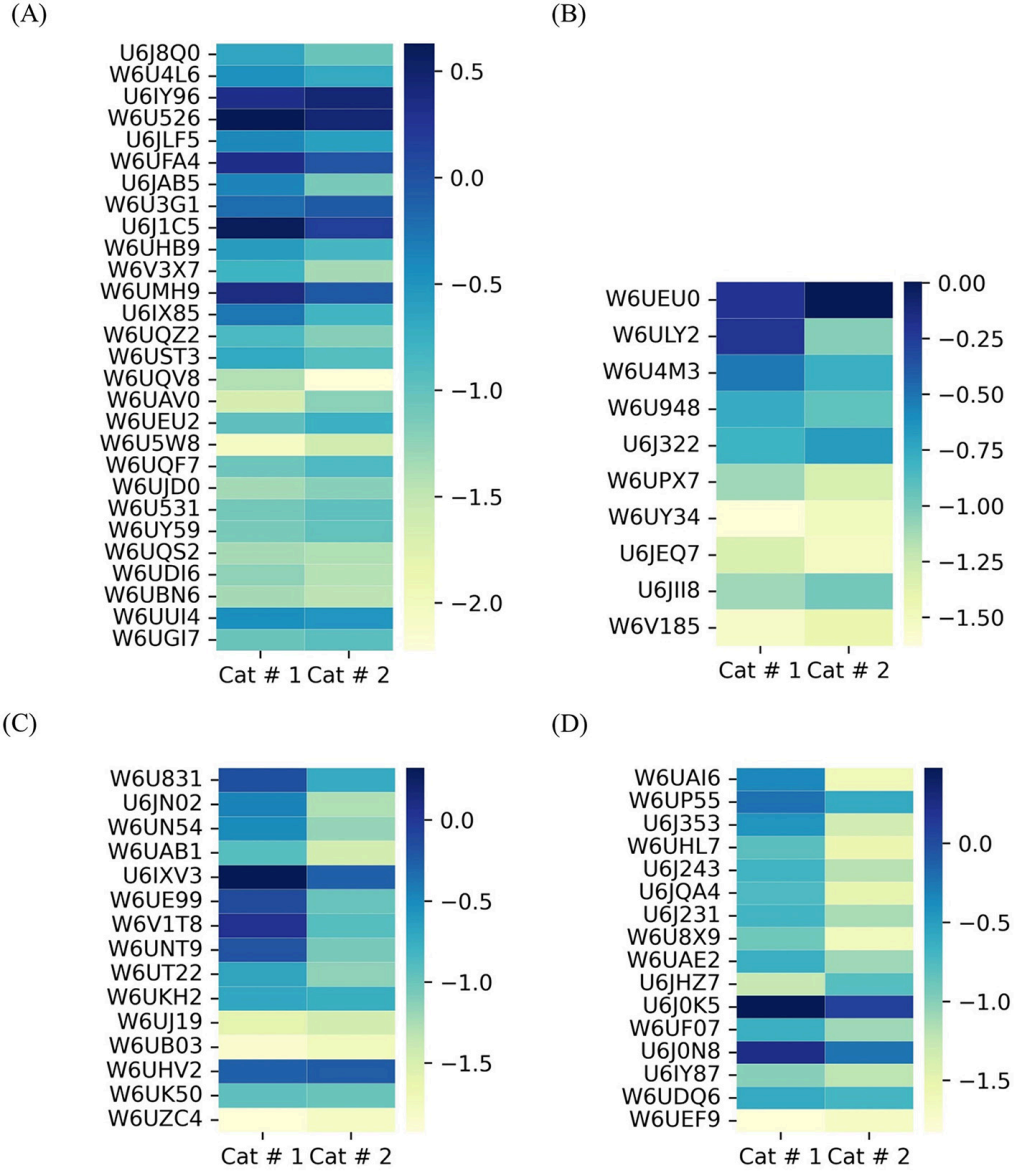
Heatmap of shared metacestode proteins classified by clusters. The proteins are organized from top to bottom according to X^2^ test results. To visualize low proportions, the log_10_ value was applied. **(A)** Cytoskeletal proteins and microfilaments. *p* < 0.001: from U6J8Q0 to W6UEU2; *p* < 0.05: W6U5W8 and W6UQF7; and without significant differences: W6UJD0 to W6UGI7. **(B)** Nuclear proteins. *p* < 0.001: from W6UEU0 to W6U4M3; (*p* < 0.05): from W6U948 to W6UPX7; and without significant differences: from W6UY34 to W6V185. **(C)** Proteins involved in glucose metabolism. *p* < 0.001: from W6U831 to W6UT22; and without significant differences: from W6UKH2 to W6UZC4. **(D)** Mitochondrial proteins. *p* < 0.001: from W6UAI6 to U6J0N8; *p* < 0.05: U6IY87 and W6UDQ6; and without significant differences: W6UEF9.

A highly abundant protein family displayed a higher proportion in 14 out of 28 proteins in Cat # 1 and in 6 out of 28 proteins in Cat # 2 (Figure 7A, Supplementary Table 3). The tegumental protein, a pleiotropic intermediate filament that binds calcium, had a higher relative proportion in Cat # 2 (Supplementary Table 3).

###### 3.3.1.1.4 Nuclear protein

They are related to DNA repair and mitosis, and they showed higher relative abundance in Cat # 1. In contrast, a higher relative proportion of a ubiquitin-conjugating enzyme was observed in Cat # 2 (Figure 7B, Supplementary Table 3).

##### 3.3.1.2 Cellular metabolism

###### 3.3.1.2.1 Glucose metabolism

A significantly higher relative abundance of proteins involved in glucose metabolism was observed in Cat #1 (Figure 7C). Supplementary Table 3 highlights differences in the relative abundance of proteins associated with glycogen synthesis and degradation.

###### 3.3.1.2.2 Mitochondrial metabolism

These proteins were also predominant in Cat # 1, with 14 out of 16 proteins being more represented in this sample (Figure 7D, Supplementary Table 3).

###### 3.3.1.2.3 Lipid metabolism

**Figure 8:**
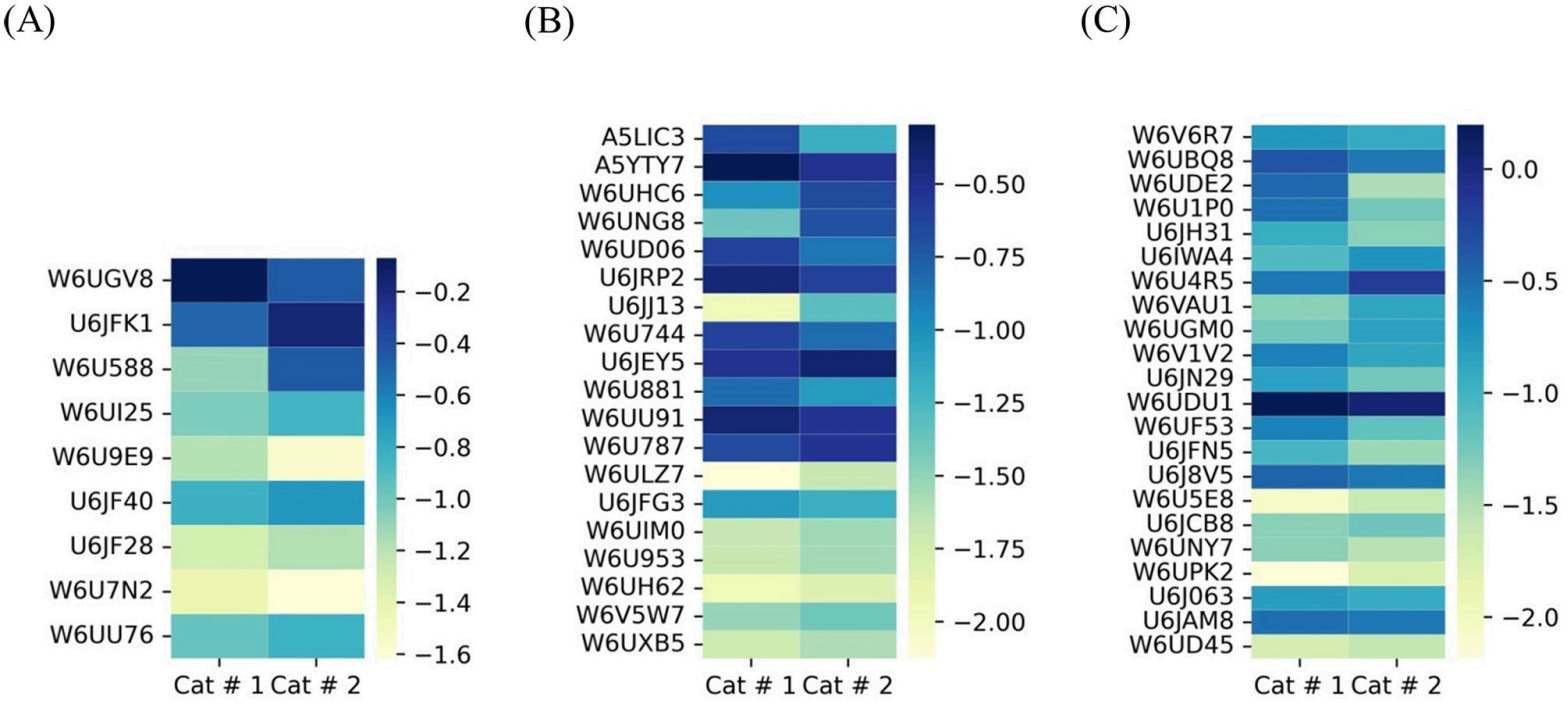
Heatmap of shared metacestode proteins classified by clusters. The proteins are organized from top to bottom according to X ^2^ test results. To visualize low proportions, the log_10_ value was applied. **(A)** Proteins involved in lipid metabolism. p <0.001: from W6UGV8 to W6UI25; *p* < 0.05: W6U9E9 and U6JF40; and without significant differences: U6JF28 to W6UU76. **(B)** Proteins involved in protein synthesis. *p* < 0.001: from A5LIC3 to U6JEY5; *p* < 0.05: W6U881 to W6ULZ7; and without significant differences: from U6JFG3 to W6UXB5. **(C)** Proteins involved in proteolysis. *p* < 0.001: from W6V6R7 to U6JFN5; *p* < 0.05: U6J8V5 and W6U5E8; and without significant differences: from U6JCB8 to W6UD45.

Proteins involved in lipid metabolism were relatively more abundant in Cat # 2 (Figure 8A), although W6UGV8, a fatty acid binding protein, was higher in Cat # 1 (Supplementary Table 3).

###### 3.3.1.2.4 Protein metabolism

It was analyzed by separating proteins involved in synthesis (Figure 8B) and proteolysis (Figure 8C). Cat # 1 showed a higher relative abundance of proteins related to protein folding, isomerization, and degradation. In addition, Ndr (αβ) hydrolase may be involved in parasite nutrition, whereas Cat # 2 showed a higher relative abundance of proteins involved in protein synthesis and post-translational modifications (Supplementary Table 3).

##### 3.3.1.3 Proteins involved in cellular signals

**Figure 9:**
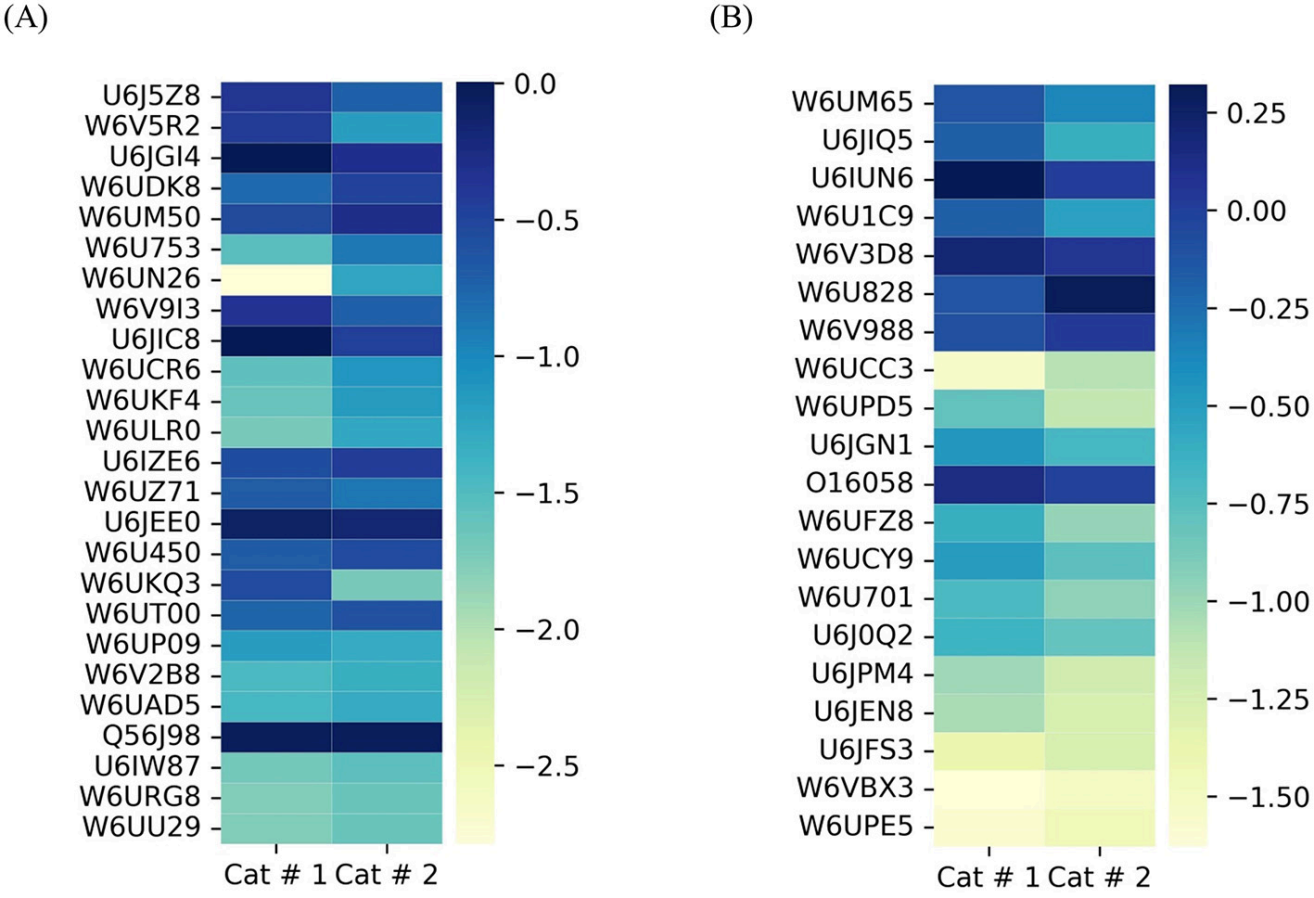
Heatmap of shared metacestode proteins classified by clusters. The proteins are organized from top to bottom according to X^2^ test results. To visualize low proportions, the log_10_ value was applied. **(A)** Proteins involved in signal transduction. *p* < 0.001: from U6J5Z8 to W6ULR0; *p* < 0.05: from U6IZE6 to W6UT00; and without significant differences: W6UP09 to W6UU29. **(B)** Proteins involved in oxide-reduction and detoxification. *p* < 0.001: W6UM65 to W6U701; *p* < 0.05: U6J0Q2 to U6JEN8; and without significant difference: U6JFS3 to W6UPE5.

Twenty-five proteins were associated with signal transduction. Cat # 1 showed a higher relative abundance of 14-3-3 proteins, whereas Cat # 2 had a higher relative abundance of ras-proteins and a protein involved in isoprenoid synthesis (Figure 9A, Supplementary Table 3). Neurotransmission-related signaling was represented by a TPM domain-containing protein, the acetylcholine-gated channel (UniProt W6UCC4), which showed higher relative abundance in Cat # 2 (Supplementary Table 3).

##### 3.3.1.4 Defense mechanisms

Defense mechanisms related to redox balance and detoxification are shown in Figure 9B. Cat # 1 had a higher relative abundance of proteins involved in detoxification and glutathione transfer. In contrast, antioxidant enzymes, such as cytoplasmic superoxide dismutase and glutathione peroxidase, were relatively more abundant in Cat # 2 (Supplementary Table 3).

##### 3.3.1.5 Antigenic proteins

**Figure 10:**
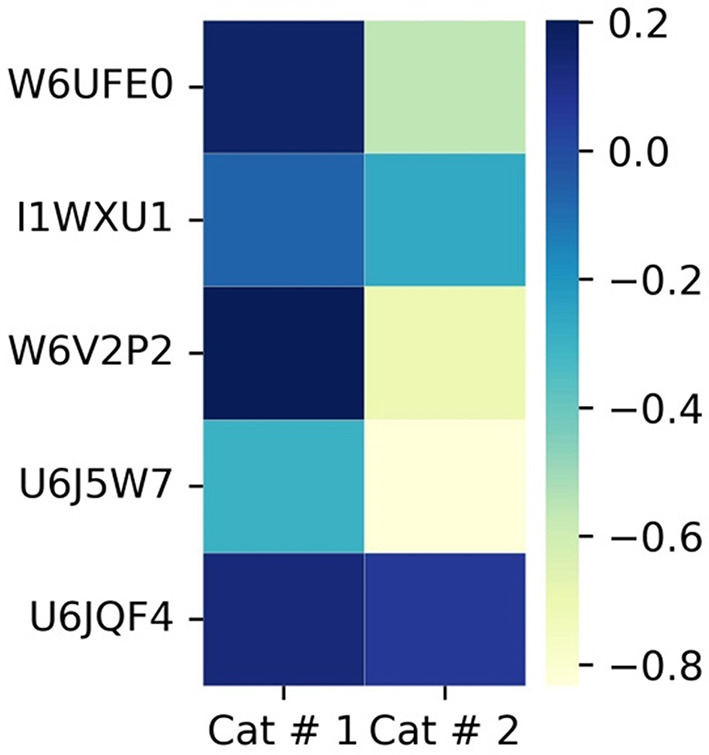
Heatmap of shared antigens in metacestodes. The proteins are organized from top to bottom according to X ^2^ test results. To visualize low proportions, the log_10_ value was applied. *p* < 0.001: from W6UFE0 to U6J5W7; and *p* < 0.05: U6JQF4.

All five antigenic proteins used as serological markers of infection (Figure 10, Supplementary Table 3) exhibited significantly higher proportions in Cat # 1.

##### 3.3.1.6 Miscellanea

Uncharacterized proteins were also identified (Supplementary Table 3).

#### 3.3.2 Non-shared proteins

**Figure 11:**
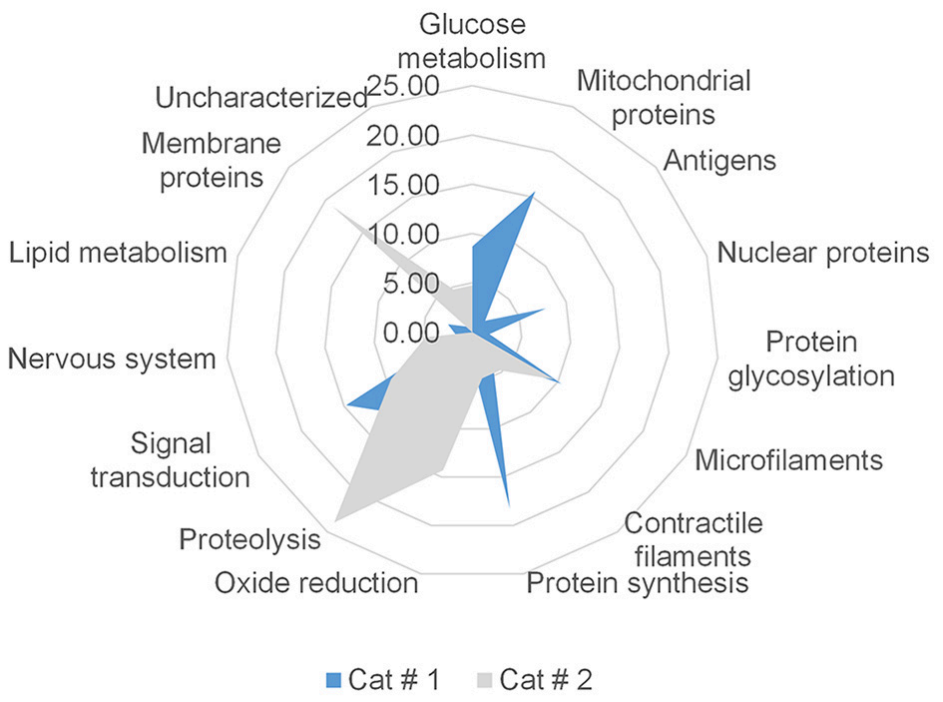
Proportional distribution of non-shared proteins based on their quantification in each cluster. Proteins were grouped according to localization or function, and proportional values were calculated according to the number of proteins belonging to each cluster. Cat # 1 has a total of 115 non-shared proteins, and Cat # 2 has 21 proteins. The X^2^ was *p* < 0.001 in all protein groups.

A difference in the number of non-shared proteins between samples was observed. The number of expressed proteins according to metabolic clusters of non-shared proteins is shown in Figure 11. Both metacestodes presented different proportional cluster distributions, and the X^2^ test revealed significant differences (*p* < 0.001) between both samples within each cluster.

##### 3.3.2.1 Cat #1

This sample exhibited a greater number of proteins associated with anabolic processes, particularly those involved in ATP production via glycolysis, the mitochondrial carbon cycles, and the respiratory chain. Anabolic pathways related to protein and lipid metabolism were also prominent, including lipids as estradiol 17-β-dehydrogenase (UniProt W6U2I8) and endoglycoceramidase (UniProt W6UMJ6). Enhanced mitochondrial activity in the non-shared proteins was represented by mitochondrial superoxide dismutase (UniProt U6J2V9), which is involved in quenching for free radicals. Several proteins involved in cellular signaling were also detected, including components of the Ras signaling pathway and a protein associated with epidermal growth factor (EGF) receptor (UniProt W6UK03). Additionally, this sample showed a higher number of proteins related to the nervous system. Nuclear proteins included those involved in DNA repair, such as histone H2B (UniProt U6JCI6), and transcriptional regulators, such as transcriptional-activator protein Pur-alpha (UniProt W6UTT2). The contractile filaments included two troponins and one tropomyosin (UniProt W6V918, W6UL31, and U6IYB3). Moreover, a tegumental protein isoform (UniProt U6JFD4), characterized as a tegumental antigen, was found. Notably, Cat # 1 expressed two isoforms of egg antigen (UniProt U6JBW8 and W6URS7).

##### 3.3.2.2 Cat # 2

This Cat #2 sample contained a higher number of membrane proteins and proteins associated with redox processes, with a bias toward peroxidation and proteolysis. For protein synthesis, a peptidylprolyl isomerase (UniProt C6ZI52) was identified. Protein involved in glycolysis was the ATP-dependent 6-phosphofructokinase (UniProt W6UHP3), and for the contractile filament was a kinesin-like protein (UniProt U6J6Z7). In this sample, membrane protein included those directly involved in inflammatory processes (UniProt W6UE44) and DUF5734 domain-containing protein, rich in heparan sulfate (UniProt W6V2K4).

### 3.4 Analysis of percent identity

The most abundant protein isoforms were found in dynein light chain (nine proteins), annexin (six proteins), and titin (five proteins). Isoform analysis revealed a high degree of amino acid sequence diversity; only a few dynein light chains had ~80% identity.

Lack of identity between some compared proteins was observed in several cases, including expressed protein (UniProt U6J5W7 and U6JME9), peroxidasin (UniProt W6UL37 and W6UBP8), tegumental protein (UniProt W6U648), peptidyl-prolyl cis-trans isomerase (UniProt C6ZI52), heat shock protein (UniProt W6UKQ3 and W6U9X5), and dynactin subunit (UniProt W6V5N2 and U6IUZ5).

## 4 Discussion

The infective larval characteristics of *E. granulosus* are difficult to study in the laboratory due to differences between laboratory animals and the natural intermediate host, as well as ethical concerns related to studying parasites in dogs and the high economic costs associated with using livestock. Epidemiological studies are also limited by the need for many infected animals or human samples. Naturally infected *Felis catus* with *E. granulosus* presents a different pathogeny compared to canids, likely due to differences in intestinal microbiota (54), which are a consequence of the differences in dietary preferences and intestinal enzyme profiles (55). This study presents the first report of research conducted on metacestode parasite material from *Felis catus* FIV-free based on clinical (46, 47) and confirmed by the LC-MS/MS method.

This study investigated *in vitro* pe behavior, cytogenetics, and differences in the expression of proteins associated with different clusters involved in parasite–host interaction.

### 4.1 Findings

#### 4.1.1 Parasite culture

Primary isolated cell culture constitutes a laboratory tool for studying the parasite's ability to survive and respond to the host's molecular stimuli, supporting efficient cell mitosis. The pe *in vitro* development mimics the sequence of events leading to the formation of the laminar membrane, which provides structural support for stem cells and facilitates communication with the host. *In vitro* pe development showed the four described stages in culture. Moreover, *in vitro* pe culture did not evolve to parasite vesicle or embryo formation.

The primary culture of isolated cells or pe in the present study exhibited similar behavior to that observed in previous studies from bovine pe (33), suggesting that the *in vitro* cellular response is independent of the host's origin. Additionally, tissue culture approaches did not yield conclusive results regarding parasite vitality or aggressiveness.

#### 4.1.2 Cytogenetic studies

There are very few published images of *E. granulosus* s.l chromosomes. Cytogenetic analysis was conducted on primary cell culture and revealed the presence of small chromosomes, approaching the resolution limit of transmission light microscopy, and only 15% of metaphases repeated the diploid number of 18 chromosomes.

#### 4.1.3 Protein relative abundance

We identified 562 total proteins in Cat # 1 and 533 in Cat # 2, and host-derived contaminant proteins accounted for 40–54% of the total proteins identified.

Ferritin was the most predominant protein in both samples, with a higher proportion in Cat # 2. Ferritin is known for its role in inflammatory response protein, and it has been identified at high levels in metacestodes by other authors (56). In the sample from Cat #1, glutaredoxin 3 was also detected, which plays a role in iron redistribution. The other protein with higher relative abundance was the tegumental, which exhibited a higher relative proportion in Cat # 2. In addition, a basement membrane rich in heparan sulfate protein (UniProt W6V2K4), which may inhibit cell migration (57), was detected exclusively in Cat # 2.

Antigenic proteins were relatively more abundant in Cat # 1, and the major egg antigen was exclusively detected in this sample. Regarding metabolic activity, proteins related to cellular architecture and cytoskeleton, such as dynein light chain and tubulin, which are essential for singular syncytial formation (58), ensure the ATP distribution (59) and allow cell division (60), were relatively more abundant in Cat #1. Additionally, Cat # 1 metacestode has a higher probability of being metabolically more active, producing more ATP and NADH. In concordance, mitochondrial proteins associated with carbon and electron cycles, essential for growth and development (61), also had a higher proportion in Cat #1, and mitochondrial superoxide-dismutase was found exclusively in this sample. Additionally, this sample had a higher relative abundance of ATP-dependent proteins, such as contractile filaments, myosin, and tropomyosin (62). Furthermore, this sample showed a higher relative proportion of a fatty acid-binding protein with 100% amino acid identity with EgFABP2 (63, 64) and 17 beta-dehydrogenase found only in Cat # 1.

Cat # 2 showed a higher relative abundance of other lipid binding proteins, enzymes associated with proteolysis, and cupin-2 protein, involved in isoprenoid biosynthesis, which was detected exclusively in this sample. Isoprenoid biosynthesis is essential for the correct localization of Ras proteins, which also had a higher proportion in Cat # 2. Moreover, this sample had a higher relative abundance of contractile filaments that require less energy for their function, such as paramyosin and tropomodulin. The high peroxidative status of Cat # 2 was reflected by an increased proportion of ferritin and proteins associated with peroxidation and the higher proportion of cytoplasmic superoxide dismutase. In contrast, proteins related to detoxification were less represented in Cat # 2. A higher proportion of specific calcium binding protein was also observed in Cat # 2, potentially indicating tissue degeneration (65) and an increase of membrane permeability.

### 4.2 Contrast with previous results

#### 4.2.1 Cell culture

In earlier studies, we obtained a cell line from the pe of *E. granulosus* ss/G1 that behaved similarly *in vitro* to the isolated cells described in this study, the EGPE cell line (33). These cells were obtained after enzymatic treatment with papain and an extended culture period. They expressed *E. granulosus* antigens (31) and formed cell cyst colonies in culture (33). Other authors performed primary cell cultures of cells from *E. granulosus* ss/G1 using trypsin, without the addition of HF to the primary culture (66).

Primary cultures of isolated cells exhibited improved proliferation following enzymatic activation, likely due to the physiological relevance of parasite–host interaction (67). The final sequence of events leading to the formation of the parasitic cyst has not yet been achieved in culture with *E. granulosus*, in contrast to *E. multilocularis*, where this has been successfully established (68). Moreover, Kaethner et al. succeeded in obtaining vesicle formation when cells from proliferative membranes were co-cultured with RH cells (69).

#### 4.2.2 Cytogenetics

There are very few published images of *E. granulosus* s.l chromosomes (70). In the present study, only 15% of metaphases displayed the reported diploid number of 18 chromosomes, as previously described in *E. multilocularis* (71). The predominant finding was heteroploidy across various metaphases, characterized by a high frequency of small chromosomes. These findings are consistent with previous reports of increased heterochromatin content and telomere attrition (72). Similar chromosome distribution, with telomere attrition and pronounced heteroploidy, was observed in metaphases from the EGPE cell line established by our group (33). Only a limited number of studies have addressed the *E. granulosus* karyotype, but we found no reports directly comparable to our observations (71, 73). In *E. granulosus*, as in other organisms with holocentric chromosomes, entire chromosomes may undergo fusion. In nematodes, chromosomal fusion and fission events can result in acentric or multicentric chromosomes, leading to genomic instability (74). Moreover, centromeric fusions are considered drivers of genomic and karyotypic instability, and frequent fusion events involve specific telomere sequences. Moreover, fusion events increased instability associated with holocentric chromosomes (75). Holocentric chromosomes and telomeric associations, such as those observed in our sample, have been reported in other nematodes. The rapid formation of new telomeres at breakpoints facilitated karyotype changes through chromosome fissions and rearrangements (76).

Examples of genome flexibility in response to adaptation to the host environment have been reported (77, 78). Tetraploid *C. elegans* have been obtained in a laboratory through mutation of the rec-8 gene, causing defects in chromosome segregation during meiosis (79). These tetraploid worms exhibited increased sensitivity to chemotherapeutic agents and a shorter lifespan (80). In tissue culture, polyploid cells derived from *E. granulosus* pe, EGPE cells, survived and proliferated; however, they exhibited a reduced cell size (33).

#### 4.2.3 Proteomic studies

Several previous studies have applied LC-MS/MS to *E. granulosus*. In HF from the human host metacestode, 63 proteins were identified, with the notable abundance of antigenic proteins and enzymes involved in glycolysis in fertile cysts (81). Comparative LC-MS/MS analysis of HF, from different intermediate hosts and metacestodes of varying fertility, identified 27 out of 44 proteins as shared among the parasites (82). Additionally, MALDI-TOF-MS analyses of HF from naturally infected sheep (CE1 and CE2) identified 78 proteins, 40 of which were of parasite origin. These included AgB and Ag5, enzymes involved in glycolysis, proteolysis, lipid metabolism, and detoxification, and antigen proteins were greater in the early stage (83). Other authors identified 1,588 proteins from pe *E. granulosus s*.s. from sheep, including the major antigen protein, which showed high amino acid sequence homology to that of *E. multilocularis* (84). A proteomic analysis of *E. granulosus* metacestode components identified 94 proteins in pe, 25 in the germinal layer, and 20 in HF (85). A proteomic study conducted after *in vitro* induction of pe strobilation identified 365 proteins, of which 75 were differentially expressed depending on the stimulus, and 51 were exclusively expressed at specific experimental stages (86). Another proteomic analysis of vesicles released from pe and metacestode in culture identified several proteins, including gelsolin, Hsp-70, traspanin, and 14-3-3 protein, while a total of 298 proteins were detected in pe (87). Additionally, LC/MS-MS analysis of experimental mice infected with *E. granulosus* s.s. revealed temporal differences in protein expression: glutathione transferase was more abundant during late infection-time stages, whereas heat shock proteins were more prominent in the early infection (88). The iTRAQ-base proteomic profiling across different life cycle stages of *E. granulosus* revealed that oncospheres express higher abundance of proteins involved in metabolic pathways, heat shock proteins, and contractile filaments associated with low-energy requirement, compared to worm or pe stages (89).

Analyzing HF from bovine lung fertile hydatid cyst from *E. granulosus* and *E. ortleppi* and among the identification of 280 proteins from *E. granulosus* and 251 proteins from *E. ortleppi*, the authors found proteins involved in adhesion, extracellular organization, development regulation, signaling transduction, and enzyme activity (90).

### 4.3 Implications

#### 4.3.1 Epidemiological implications

*Felis catus* is an accidental intermediate host with epidemiological relevance linking the wild environment with the domestic one. Infected *Felis catus* may become part of the parasite life cycle if ingested by wild canids. This could lead to alterations in parasite aggressiveness, potentially transmitted through the oncosphere to domestic settings. In addition, cats may function as passive mechanical vectors by transporting oncospheres on their limbs and fur to different environments, thereby facilitating transmission to selective populations and contributing to distinct epidemiological patterns, as reported in earlier studies. In Turkana, females were found to be infected with a different *E. granulosus* genotype compared to males (5). Moreover, changes in the intermediate host could change the parasite's infective genius (39). Cats with CE may serve as epidemiological sentinels.

#### 4.3.2 Clinical implications

As mentioned in the introduction, advanced abdominal CE in cats has been reported in various geographic regions (16, 40–47). Differential diagnosis with other abdominal pathologies must be conducted in the veterinary clinic (91).

#### 4.3.3 Biological implications

*Ex vivo, in vitro*, and molecular analysis may help determine whether the metacestode carried by *Felis catus* has distinctive *in vitro* behavior compared to those from other studied hosts. This study is the first to report *ex vivo, in vitro*, cytogenetic, and entire vesicle proteomics analysis of *E. granulosus* G1/ss metacestode from two unrelated *Felis Catus*.

##### 4.3.3.1 *In vitro* behavior and karyotype

Biological plasticity enables cells to rapidly adapt to new environmental conditions (92). Few studies in the literature reported *ex vivo* and *in vitro* cultures of *E. granulosus* pe, and cytogenetics and karyotype data remain poorly described. In this study, we performed pe cell cultures and karyotyping for the second time and obtained results consistent with previous observations (33). The most remarkable difference was that heteroploidy and termini-termini telomere attrition were less frequent in primary culture than in the EGPE cell line. These modifications may reflect cellular adaptation to a new environment, possibly similar to the transition that occurs when a parasite shifts from an intermediate to a definitive host (93–95). Genome instability and chromosomal rearrangement may lead to DNA variation, which could explain the observed variability in genetic polymorphisms such as nad, cox, and AgB (2–5, 23) across different genotypes. Moreover, these chromosomal rearrangements could promote genetic variation in embryos, even under asexual reproduction.

##### 4.3.3.2 Proteomic analysis

This is the first LC-MS/MS report about the protein expression of two metacestode vesicles from *Felis catus*. Expression of proteins may depend on the stage of larva (active or inactive), varying according to early or late metacestode vesicle life or parasite–host relation. We found a higher proportion of proteins involved in energy and glucose metabolism, as well as a high proportion of AgB and Ag5 in Cat # 1, indicating active metabolism in the parasite (90, 96). In addition, the expression of the major egg antigen was exclusively detected in Cat # 1. Failure in the vaccination schedule with EG95 could be attributed to different metacestode protein expression (97). In this study, metacestodes from Cat # 2 did not express the major egg antigen at detectable levels but expressed a rich heparan sulfate protein, which inhibits cell migration, and exhibited a low relative abundance of other *E. granulosus* antigens, modulating host response.

### 4.4 Limitations

This study was conducted using only two vesicles from two unrelated FIV-negative cats. Due to the limited amount of starting material, the study lacks technical replicates for the LC-MS/MS analysis, and transcriptomic analysis was not performed. The methodology used requires at least 30 μg of protein per sample, and individual vesicles did not yield enough protein to allow duplication. Additionally, other vesicles might present distinct protein profiles with intermediate values. Therefore, we focused on individual vesicle analysis, rather than pooling, to capture potentially meaningful biological variability.

In this study, we present results from only two vesicles, which shared the majority of identified proteins but displayed differences in their relative abundances. These differences may reflect distinct metacestode behaviors, and we considered that even a single highly viable vesicle may be sufficient to perpetuate the infection or to infect the definitive host.

Due to the limitations in obtaining isolated parasite cells capable of maintaining typical *in vitro* behavior and the inability to perform karyotyping in organ cultures, cytogenetic analysis was performed on only one sample. However, our previous findings confirm that this chromosomal behavior is consistent in culture and likely represents an early cellular adaptation to altered environmental conditions (92).

### 4.5 Future research directions

Imaging techniques are not useful for detecting early recurrence or determining the initial stage of the infection, which is more sensitive to antiparasitic treatment. Other authors have investigated native antigens (98) and nuclear receptors involved in parasite–host communication (99), as well as stage-specific serological markers (100).

Infection recurrences are often associated with treatment failure (101) and incomplete implementation of epidemiological control measures. Additionally, parasite genius and intermediate host resistance constitute factors contributing to the infection success. In addition to the main importance of the parasite transition from larval to adult stage being related to host age (102), there is still no consideration of parasite fitness.

Future directions must cover clinical and epidemiological controls of CE in *Felis catus* and controls on CE infection in humans and animals in contact with CE-infected cats. Therefore, it is necessary to develop serological markers for CE infection that reflect parasite metabolic activity, which is not always correlated with imaging findings.

In addition, it is important to develop pharmacological drugs tailored to the parasite's metabolic status for the treatment of both humans and cats.

## 5 Conclusion

Our results show, for the first time, the *in vitro* development, cytogenetic characterization, and molecular profiling by LC-MS/MS of CE developed in *Felis catus*. The metacestodes exhibited molecular characteristics similar to those observed in humans and ungulate hosts, expressing the same antigens and demonstrating active metabolism, *in vitro* cell proliferation, and stimulus response. Parasite-cell metaphase and karyotype characteristics could help in understanding the rapid molecular modifications produced in the parasite by the new host or environment. Indeed, the final metaphase resolution could lead to the success or failure of the infection. Molecular analysis of the metacestodes may provide insights into the parasite's local aggressiveness or its potential to develop into the worm stage in the definitive host. Among the diverse protein isoforms identified, many canonical proteins were expressed in more than one metacestode, with varying relative abundance, as shown by heatmap analysis and statistical evaluation. These differences may be associated with host–parasite relationship.

In this study, the relative proportion of 225 shared proteins between cysts contributed to the characterization of cyst behavior. Proteins were classified by functional cluster, and for a relative abundance study, heatmaps were used, along with the X^2^ test, because differences in color intensity are difficult to appreciate when extreme values are plotted. Notably, Cat # 2 may exhibit a slower metabolism than Cat # 1, potentially eliciting a weaker host immune response while possibly generating more free radicals, increasing local inflammation. Moreover, rich heparan sulfate protein found in Cat #2 could indicate less local invasiveness. These findings support the idea that the host's antibody reactivity characteristics (affinity, specificity, and sensitivity) can serve as an indicator of parasite vitality, with implications for local dissemination and completion of the parasite's life cycle.

## Data Availability

The mass spectrometry proteomics data have been deposited to the ProteomeXchange Consortium via the PRIDE partner repository with the dataset identifier PXD067276.
