# Supplementary material for: First comparative proteomic and in vitro behavioral study of Echinococcus granulosus metacestodes in Felis catus
Source: Front Vet Sci. 2025 Sep 2;12:1546420. doi: 10.3389/fvets.2025.1546420 (PMC12436101; doi:10.3389/fvets.2025.1546420)
Supplement: Supplementary file 3 [file Table_3.pdf]

<u>Supplementary Table 3</u>. Proportion of proteins relevant to parasite metabolism. Statistical significance assessed with chi-square test (*p< 0.001, °p< 0.05).

UniProt	**Protein name**	Cat # 1	Cat # 2
	**Contractile filaments**		
	**High energy requirement**		
W6UFT0	tropomyosin	0.2271*	0.0731
W6UWX8	tropomyosin	0.9080*	0.1528
W6UYQ6	myosin	0.7914*	0.1221
W6UMY5	myosin	0.3055*	0.1632
	**Low energy requirement**		
W6UDZ9	paramyosin	0.9745	3.7391*
W6UJP6	paramyosin	0.0170	0.1243*
W6UKX8	tropomodulin	0.1942	0.2614°
	**Cytoskeletal proteins and microfilaments**		
W6UAV0	actin-related protein 3	0.0217	0.0609*
W6UEU2	spectrin alpha chain	0.1038	0.1707*
W6UQF7	spectrin beta chain	0.0867	0.1315°
W6U5W8	dynamin -GTPase	0.0089	0.0245°
W6U648	tegumental protein	23.0850	43.2911*
	**Nuclear proteins**		
W6ULY2	histone H4	0.6049*	0.0916
W6U948	transgelin	0.1721°	0.1193
U6J322	ubiquitin-conjugating enzyme	0.1558	0.2096°
W6UPX7	protein SET	0.0779°	0.0486
	**Glucose metabolism**		
U6IXV3	fructose bisphosphate aldolase	2.0986*	0.5504
W6V1T8	glyceraldehyde 3-phosphate dehydrogenase	1.1146*	0.1221
W6UT22	UTP-glucose-1-phosphate uridylyl transferase	0.2038*	0.0731
	**Mitochondrial proteins**		
	**Carbon cycle**		
U6J0K5	cytrate synthase	3.0069*	1.1834
U6J0N8	ornithine aminotransferase	1.7202*	0.5944
	**Respiratory chain**		
U6IY87	Complement component-1-Q subcomponent binding	0.0991°	0.0609
W6UF07	cytochrome-b-c1 subunit 7	0.2320*	0.0813

UniProt	**Protein name**	Cat # 1	Cat # 2
	**Protein synthesis**		
A5YTY7	calreticulin	0.5041*	0.3003
W6UNG8	murino globulin-2	0.0417	0.2002*
U6JRP2	peptidyl-prolyl-- cis trans-isomerase	0.3754*	0.2441
W6UU91	peptidyl-prolyl-- cis trans-isomerase	0.3924°	0.2922
W6ULZ7	PDZ-domain containing protein	0.0075	0.0207°
U6JJ13	threonine-tRNA ligase	0.0114	0.0486*
U6JEY5	ADP-ribosylation factor	0.3055	0.4111*
	**Proteolysis**		
W6U4R5	cystatin domain-containing protein	0.2691	0.6785*
W6UDU1	ubiquitin	1.5723*	1.1429
W6UF53	Ndr (alpha,beta hydrolase)	0.2367*	0.0697
W6U5E8	Huntingtin-interacting protein	0.0086	0.0239°
	**Signal transduction**		
U6J5Z8	14-3-3 protein	0.4239*	0.1836
U6JGI4	14-3-3 protein	1.0113*	0.4911
U6JEE0	14-3-3 protein	0.8084°	0.6785
W6UCR6	Ras-related protein Rab-27A	0.0270	0.0769*
W6UKF4	small monomeric GTPase	0.0245	0.0697*
W6ULR0	Ras-related protein Rab-7a	0.0194	0.0540*
U6IZE6	cupin-2	0.2691	0.3621°
	**Neurotransmitter system**		
W6UCC4	TPM-domain-containing protein (acetylcholine channel)	0.0086	0.0239°
	**Oxide reduction and detoxification**		
	**oxide -reduction**		
U6JNP4	ferritin	59.4139	83.1864*$^{+}$
U6IUN6	thioredoxin-dependent peroxiredoxin	2.0986*	1.0095
W6U828	thioredoxin domain-containing protein	0.7501	2.0395*
W6V988	superoxide dismutase	0.7914	1.0651*
U6JEN8	glutaredoxin-3	0.0907°	0.0562
	**Detoxification**		
W6UCC3	glutathione peroxidase	0.0284	0.0813*
U6JGN1	glutathione transferase	0.3409*	0.2040
O16058	glutathione transferase	1.3555*	0.9678
W6UFZ8	glutathione transferase	0.2458*	0.1048
U6J0Q2	elongation factor 1-beta	0.2257°	0.1572
W6UCY9	metallo-beta lactamase	0.3197*	0.1695

UniProt	**Protein name**	Cat # 1	Cat # 2
	**Antigens**		
W6UFE0	Sj-Ts4	1.4702*	0.2743
I1WXU1	Ag5	0.8492*	0.5373
W6V2P2	P29	1.5968*	0.1958
U6J5W7	expressed protein	0.5041*	0.1469
U6JQF4	AgB	1.3555°	1.1429
	**Non-characterized proteins**		
W6UIJ6	uncharacterized protein	0.0602	0.0809
W6TZB8	uncharacterized protein	0.0149	0.0269
W6UBR0	uncharacterized protein (DUF221 domain-containing)	0.0604°	0.0051

+ Ferritin proportion was calculated from the total emPAI addition. Ferritin emPAI was subtracted from to calculate the other proteins proportion, as it has been described in Materials and Methods section.
