# Supplementary figures and images for: First comparative proteomic and in vitro behavioral study of Echinococcus granulosus metacestodes in Felis catus
Source: Front Vet Sci. 2025 Sep 2;12:1546420. doi: 10.3389/fvets.2025.1546420 (PMC12436101; doi:10.3389/fvets.2025.1546420)

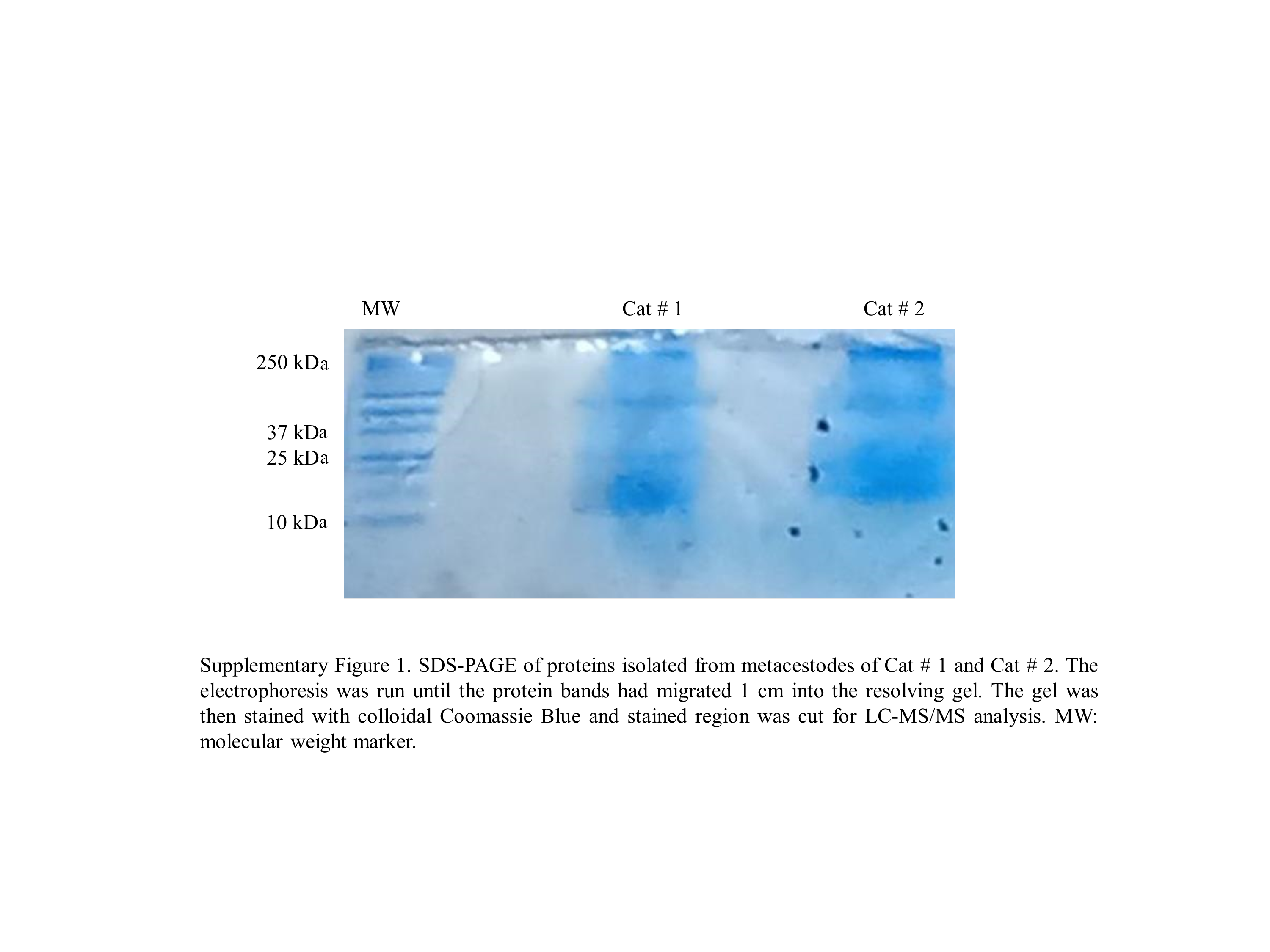

Supplement: Supplementary file 4 [file Image_1.tif]
