# Supplementary material for: First comparative proteomic and in vitro behavioral study of Echinococcus granulosus metacestodes in Felis catus
Source: Front Vet Sci. 2025 Sep 2;12:1546420. doi: 10.3389/fvets.2025.1546420 (PMC12436101; doi:10.3389/fvets.2025.1546420)
Supplement: Supplementary file 5 [file Image_2.pdf]

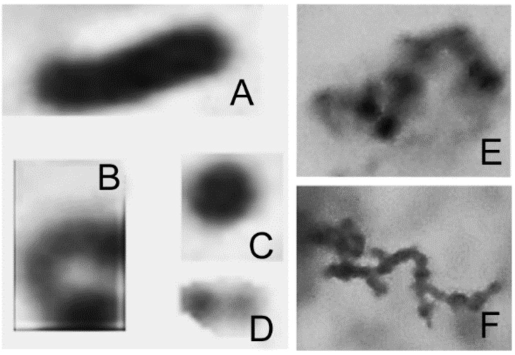

Supplementary Figure 2: Chromosomes stained with Giemsa and C bands were photographed and studied for their sizes and the position of centromere. A: Large metacentric chromosome; B: discentric – acrocentric chromosome; C: Small holocentric circle chromosome; D: Small acrocentric chromosome; E and F: Long arrays of chromosomes associated through their extreme region.
